# Label-Free Impedimetric Immunosensors Modulated by Protein A/Bovine Serum Albumin Layer for Ultrasensitive Detection of Salbutamol

**DOI:** 10.3390/s20030771

**Published:** 2020-01-31

**Authors:** Chia-Hung Lin, Ming-Jie Lin, Jie-De Huang, Yu-Sheng Chuang, Yu-Fen Kuo, Jung-Chih Chen, Ching-Chou Wu

**Affiliations:** 1 Department of Bio-industrial Mechatronics Engineering, National Chung Hsing University, No. 145, Xingda Rd., South Dist., Taichung City 402, Taiwan; dans001049@gmail.com (C.-H.L.); y4ky5k@yahoo.com.tw (M.-J.L.); jiede2jh@gmail.com (J.-D.H.); n2218152@hotmail.com (Y.-S.C.); 2 Metal Industries Research & Development Centre, Kaohsiung 811, Taiwan; Kellykuo760825@gmail.com; 3 Institute of Biomedical Engineering, National Chiao Tung University, 1001 University Road, Hsinchu 30010, Taiwan; 4 Innovation and Development Center of Sustainable Agriculture, National Chung Hsing University, No. 145, Xingda Rd., South Dist., Taichung City 402, Taiwan

**Keywords:** protein A, surface density, oriented immobilization, electrochemical impedance spectroscopy, immunosensor

## Abstract

The sensing properties of immunosensors are determined not only by the amount of immobilized antibodies but also by the number of effective antigen-binding sites of the immobilized antibody. Protein A (PA) exhibits a high degree of affinity with the Fc part of IgG antibody to feasibly produce oriented antibody immobilization. This work proposes a simple method to control the PA surface density on gold nanostructure (AuNS)-deposited screen-printed carbon electrodes (SPCEs) by mixing concentration-varied PA and bovine serum albumin (BSA), and to explore the effect of PA density on the affinity attachment of anti-salbutamol (SAL) antibodies by electrochemical impedance spectroscopy. A concentration of 100 μg/mL PA and 100 μg/mL BSA can obtain a saturated coverage on the 3-mercaptoproponic acid (MPA)/AuNS/SPCEs and exhibit a 50% PA density to adsorb the amount of anti-SAL, more than other concentration-varied PA/BSA-modified electrodes. Compared with the randomly immobilized anti-SAL/MPA/AuNS/SPCEs and the anti-SAL/PA(100 μg/mL):BSA(0 μg/mL)/MPA/AuNS/SPCE, the anti-SAL/PA(100 μg/mL): BSA(100 μg/mL)/MPA/AuNS/SPCE-based immunosensors have better sensing properties for SAL detection, with an extremely low detection limit of 0.2 fg/mL and high reproducibility (<2.5% relative standard deviation). The mixture of PA(100 μg/mL):BSA(100 μg/mL) for the modification of AuNS/SPCEs has great promise for forming an optimal protein layer for the oriented adsorption of IgG antibodies to construct ultrasensitive SAL immunosensors.

## 1. Introduction

In past few decades, immunosensors have emerged as a crucial tool for detecting various chemicals and biological compounds in applications including environmental monitoring, clinical diagnostics and food safety [1,2,3,4]. Their detection mechanism relies on the specificity and capturing ability of immobilized antibodies versus antigens. Recently, label-free techniques have been developed to directly monitor antigen–antibody interactions, such as surface plasmon resonance [5], quartz crystal microbalance [6], mass-sensitive microcantilever [7], and electrochemical impedance spectroscopy (EIS)-based immunosensors [8]. EIS-based immunosensors have attracted particular interest due to their advantages, including high sensitivity, ease of mass production and the feasibility of miniature diagnostic systems [9]. The sensing properties of label-free immunosensors are dependent on not only the amount of immobilized antibodies but also the effective antigen-binding sites (paratopes) of antibodies [10].

Electrodes have been developed with nanostructured or rough surfaces to promote the amount of immobilized antibodies on an immunosensor surface [11,12]. Gold nanostructures (AuNSs) are the most popular substrate for label-free immunosensors due to their highly electroactive surfaces, their strong adsorption ability for proteins and their ease of surface modification with thiolate molecules. AuNS deposition can be fabricated using template [13] or templateless electrodeposition [14,15]. Furthermore, the oriented immobilization of antibodies is another important strategy for improving the sensing properties of immunosensors. The amount of effective site-positioning paratopes is determined by the method of immobilization used (including adsorption, covalent binding and affinity attachment) and the surface density of immobilized antibodies. Welch et al. reviewed the method of immobilizing antibodies and the effect of paratope orientation on the sensing characterization [16]. Wang and Feng surveyed the progress on the three-dimensional oriented immobilization of proteins [17]. In principle, antibodies adsorbed on solid substrates via electrostatic, van der Waals or hydrogen-bonding interaction present a high degree of heterogeneity and randomly orientated paratopes [18]. The covalent binding method results in the immobilized antibodies having a high reproducible surface density and allows for good control over paratope orientation. Billah et al. used a 4-aminothiophenol self-assembled monolayer (SAM) to covalently link the hinge region of reduced half-antibody fragments [19]. Ferreira et al. adopted cysteamine SAM as a link to bind N-ethyl-N’-(3-dimethylaminopropyl) carbodiimide hydrochloride/N-hydroxysuccinimide (NHS)-activated antibody to control paratope orientation [20]. However, the covalent processes may induce a loss of immunorecognition activity or reduce the biological functions of antibodies, due to the hazardous chemical reactions. In contrast, affinity attachment is a more moderate and effective method to control the paratope orientation through recombinant protein A, G, A/G, L [21] or the transmembrane ZZ-L protein [22] due to the high affinity between the Fc region of antibodies and these recombinant proteins. Wang et al. directionally immobilized antibodies on interdigitated array microelectrodes with pre-adsorbed protein A (PA) to construct an impedimetric immunosensor for the detection of avian influenza virus H5N1 [23]. Hafaiedh et al. used protein G to immobilize an anti-C-reactive protein to form an impedimetric immunosensor [24]. Boujday et al. proved that the 60%-coverage PA monolayer could capture more IgG than the deglycosylated avidin and the monoclonal secondary antibody, due to the greater flexibility and better accessibility of the PA layer, which was estimated by absorption infrared spectroscopy and quartz crystal microbalance [25]. These results imply that the coverage of the PA modifying layer affects the extent of antibody adsorption. Although a moderate PA monolayer can increase the amount of captured IgG, the excessive IgG antibody may cause the hook effect to lower the immunoreactive efficiency due to steric hindrance. Furthermore, few studies have explored the method of controlling PA density and the effect of PA density on the sensing properties of impedimetric immunosensors.

This study proposes a co-immobilized method to control the coverage ratio of PA in a protein layer by mixing concentration-varied PA and bovine serum albumin (BSA). The recombinant PA and BSA can be simultaneously immobilized on the alkanethiol-modified AuNS/SPCEs, which allows antibodies to orientedly adsorb on the PA/BSA protein layer. The EIS method was used to estimate the effect of PA coverage on the amount of attached antibody. Furthermore, salbutamol (SAL), one kind of β2-agonist used in human and veterinary medicine, was used as a typical analyte to estimate the effect of effective paratopes of the adsorbed anti-SAL on the sensing properties of label-free impedimetric immunosensors.

## 2. Materials and Methods

### 2.1. Reagents and Chemicals

Recombinant PA and porcine serum were respectively purchased from BioVision (MW:39210 Da, Cat. 6500B) and Invitrogen Corp (Cat.26250084, Bibco). Highly specific sheep monoclonal anti-SAL antibody was obtained from Randox Biosciences (MAB9343). Potassium hexacyanoferrate(III) (K_3_[Fe(CN)_6_]) and potassium hexacyanoferrate(II) trihydrate (K_4_[Fe(CN)_6_]) · 3H_2_O were purchased from Showa. 3-mercaptopropionic acid (MPA), N-(3-Dimethylaminopropyl)-N′-ethylcarbodiimide hydrochloride (EDC), NHS, 2-(N-morpholino) ethanesulfonic acid (MES), gold(III) chloride trihydrate (HAuCl_4_), potassium chloride (KCl), SAL and BSA (MW:66000 Da) were purchased from Sigma-Aldrich. The 10 mM MPA solution was prepared in double distilled water. Phosphate buffer solution (PBS, pH 7.4) was prepared by 10 mM NaH_2_PO_4_ and 10 mM Na_2_HPO_4_ and used in all immune experiments. The SPCEs with a working area of 3.2 mm^2^ were obtained from Tyson Bioresearch (Taiwan). All chemicals were of reagent grade and were used without further purification. All solutions were prepared with water purified through a Milli-Q system.

### 2.2. AuNS Deposition

All electrochemical experiments were performed with an IM-6 impedance analyzer (Zahner Electrik GmbH, Germany) in a three-electrode system by using a Pt wire as the counter electrode and an Ag/AgCl electrode as the reference electrode. The bare SPCEs were first electrocleaned by sweeping the potential from 0 to 1.3 V for 20 cycles with a 0.1 V/s scanning rate in 0.1 M PBS (pH 7.0), and then oxidized at 2.0 V for 30 s to obtain more hydrophilic surfaces. After being rinsed with double distilled water, the electrocleaned SPCEs were dipped in the 8 mM HAuCl_4_ solution (pH 2.0), containing 100 mM KCl, for the two-step AuNS deposition [26]. The first step is to nucleate Au nanoparticles on the electrocleaned SPCE surface in the cyclic potential range from 0.5 to −0.5 V with a scan rate of 50 mV/s for seven cycles. The second step was performed at the half peak potential of gold reduction (~0.62 V) for 10 min to grow nanostructures on the Au nanoparticles. The AuNS/SPCEs were then rinsed with double-distilled water to remove the free ions from the electrode surface for subsequent surface modification.

The morphology of the AuNS/SPCEs was observed by field-emission scanning electron microscopy (SEM, JEOL JSM-7401F, Tokyo, Japan) with an accelerating voltage of 3.0 kV. The roughness factor of AuNS/SPCEs was defined as the ratio of the real area of AuNS to the geometric area of SPCE. The real surface area of AuNS was determined in the 50 mM HClO_4_ solution in the potential range of 0.3–1.5 V with a scan rate of 50 mV/s [26], and calculated from the oxide desorption charge density of 386 µC/cm^2^ by integrating the reductive current of the gold oxide monolayer to obtain the roughness factor [26].

### 2.3. Immunosensor Preparation

**Scheme 1:**
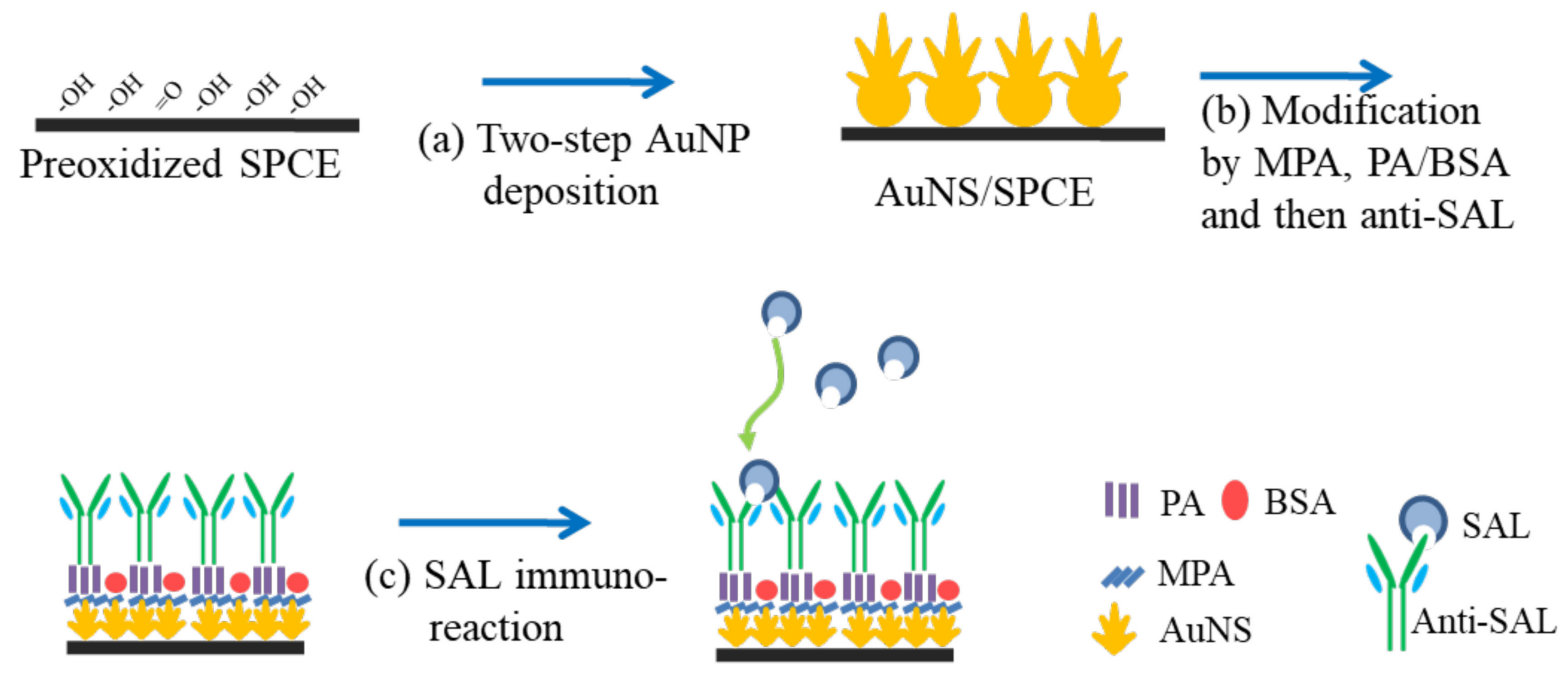
Schematic processes of the immunosensor fabrication including (**a**) two-step AuNP electrodeposition on the preoxidized SPCE, (**b**) surface modification by MPA, PA/BSA and anti-SAL, (**c**) SAL immunoreaction.

A total of 10 μL aliquot of 10 mM MPA was dripped on the surface of AuNS/SPCEs at 30 °C for 1 h in an incubator with 30% relative humidity to form an SAM, which was used as a link for the immobilization of PA and BSA. The unbound MPA molecules were removed using double-distilled water. The MPA-modified AuNS/SPCEs were soaked overnight in 1 mL 20 mM MES solution (pH 4.6), containing 30 mM EDC and 2 mM NHS, to activate the carboxyl group of MPA SAM. Subsequently, 10 μL mixture of concentration-varied PA and BSA prepared in 10 mM PBS was placed on the EDC/NHS-activated MPA/AuNS/SPCEs at 30 °C for 40 min, and then the PA-modified electrodes were dipped in PBS containing 0.05% Tween-20, called PBST, and blank PBS to remove the unbound PA and BSA. A 10 μL 0.1 mg/mL anti-SAL antibody was placed on the PA-modified AuNS/SPCEs at 30 °C for 1 h, and then rinsed with PBST and PBS. The fabricating procedures for the immunosensors are depicted in Scheme 1. After performing the oriented attachment of anti-SAL, the immunosensors were incubated with SAL samples with different concentrations prepared in PBS for 40 min at room temperature, and then cleaned with PBS. The change in the electrochemical properties of the electrode/solution interface was estimated by cyclic voltammetry (CV) and EIS.

### 2.4. Electrochemical Measurements

An equimolar Fe(CN)_6_^3−/4−^ mixture (2.5 mM) in 10 mM PBS (pH 7.4) was used as a mediator to estimate the electrochemical properties of electrodes in each modification step and quantify the immunoreaction. The CV was performed in the potential range of −0.1 V–+0.5 V at a scanning rate of 20 mV/s to evaluate the redox behavior of Fe(CN)_6_^3−/4−^ mediator on the modified electrodes. EIS measurements were performed in a frequency range of 1–100 kHz at a +0.21 V potential, added by a 5 mV amplitude sine wave. Measurement of the impedance spectra and simulation of equivalent circuits were performed using the IM-6/THALES software package.

## 3. Results and Discussion

### 3.1. Morphology and Roughness of AuNS/SPCEs

**Figure 1:**
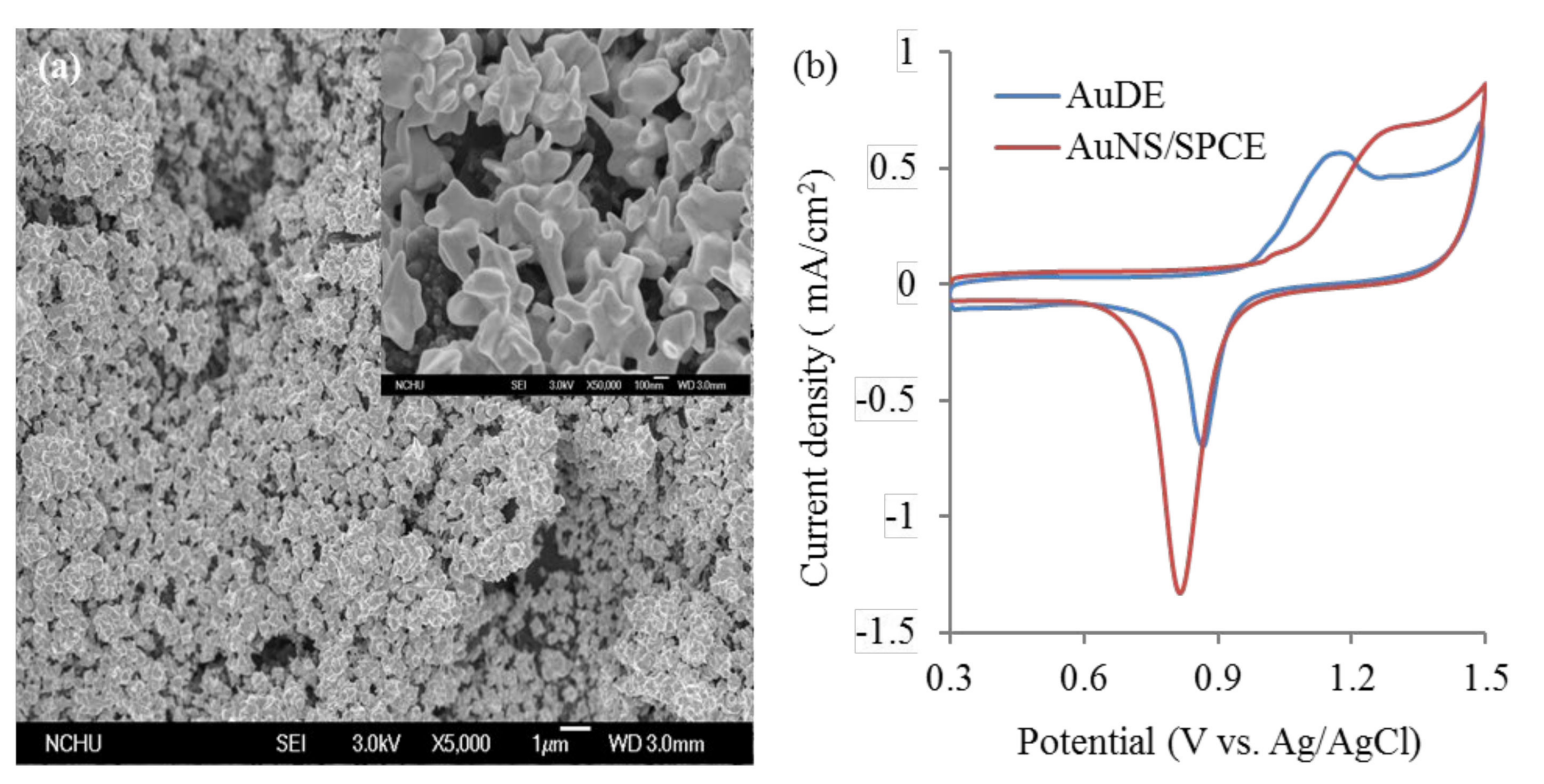
(**a**) SEM images of screen-printed carbon electrodes (SPCEs) after performing two-step gold electrodeposition. The inset shows a high magnification (× 50000) SEM image. (**b**) Cyclic voltammograms measured at a gold disk electrode (AuDE) of 1.6 mm diameter and a gold nanostructure (AuNS)/SPCE in 50 mM HClO_4_ solution with a scan rate of 50 mV/s to calculate the formation and desorption of gold oxide.

Figure 1a shows the SEM image of the morphology of AuNS/SPCEs. The result indicates that the surface of AuNS/SPCEs has well-dispersed binary gold structures with submicrometer-scaled pyramidal structures on the micrometer-scaled particles. The mechanism of two-step AuNS formation is discussed in our previous study [26]. However, the AuNS morphology in this work is slightly different from that in our previous study [26]; this inconsistency is attributed to the effect of the SPEC carbon substrate on Au particle nucleation. The highly rough AuNS helps immobilize greater bioaffinity recognition molecules or antibodies. The roughness factor of AuNS/SPCEs was evaluated by the reductive charge of gold oxide desorption, as shown in Figure 1b. By integrating the reductive current of gold oxide in the range of 0.75–1.0 V, the real surface area of the AuNS/SPCEs was 28.89 ± 0.36 mm^2^ with a corresponding roughness factor of 9.03 ± 0.11 (n = four electrodes). In contrast, the polished gold disk electrodes had a smaller roughness factor, 3.38, calculated from Figure 1b. The result suggests that the AuNS can significantly increase the surface area of SPCEs.

### 3.2. Effect of PA and BSA Mixture 

**Figure 2:**
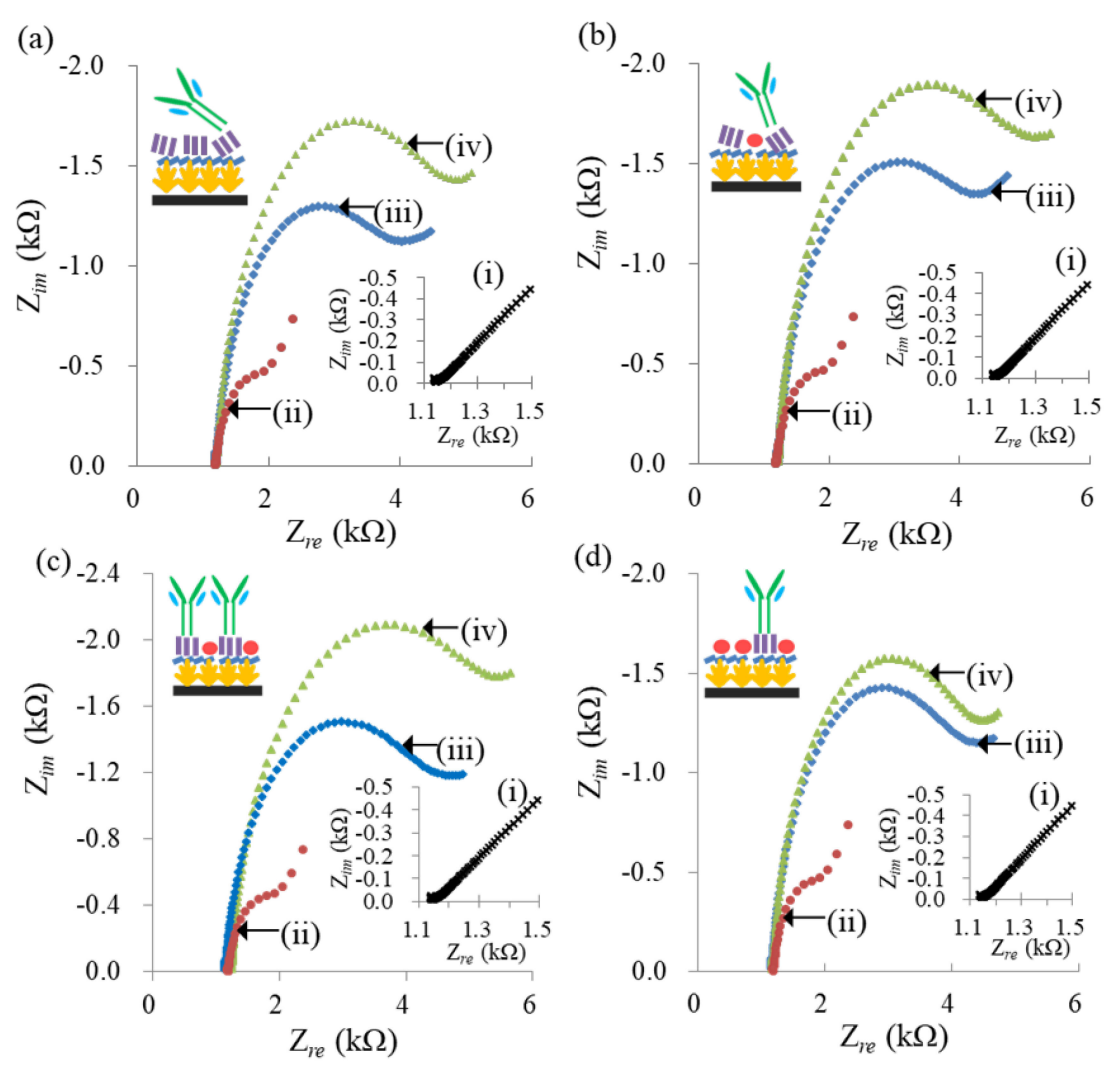
Nyquist plots obtained at the AuNS/SPCEs (i) followed by MPA modification (ii), PA:BSA immobilization (iii) and anti-SAL affinity attachment (iv) in a 10 mM PBS solution containing 100 mM KCl and 2.5 mM equimolar Fe(CN)_6_^3−/4−^. (**a**–**d**) respectively represent the electrodes immobilized by the PA:BSA mixture (μg/mL) of 100:0, 100:50, 100:100 and 100:200. The inserted schemes depict the possible arrangement and orientation of the antibodies.

Four kinds of PA and BSA mixtures with concentration (μg/mL) ratios of 100:0, 100:50, 100:100 and 100:200 were used to explore the effect of PA coverage on the amount of attached antibody. Figure 2 shows the impedance spectra measured at the AuNS/SPCEs followed by MPA modification, the immobilization of the concentration ratio-varied PA:BSA mixture, and the anti-SAL adsorption. The impedance spectra measured at the AuNS/SPCEs (curves (i) of Figure 2) exhibit a dominant linear region, implying that the redox reaction of the Fe(CN)_6_^3−/4−^ mediator at the AuNS/SPCEs is a diffusion-controlled behavior, due to its fast electron-transfer rate [26]. Following MPA modification, the Nyquist plots show a linear part at lower frequencies and a semicircle part at higher frequencies, indicative of a kinetics-controlled region. Moreover, the radius of the semicircle increased with the modification steps of the PA:BSA immobilization and the anti-SAL adsorption. The results indicate that the impedance of the solution/electrode interface is increased by the modification.

**Figure 3:**
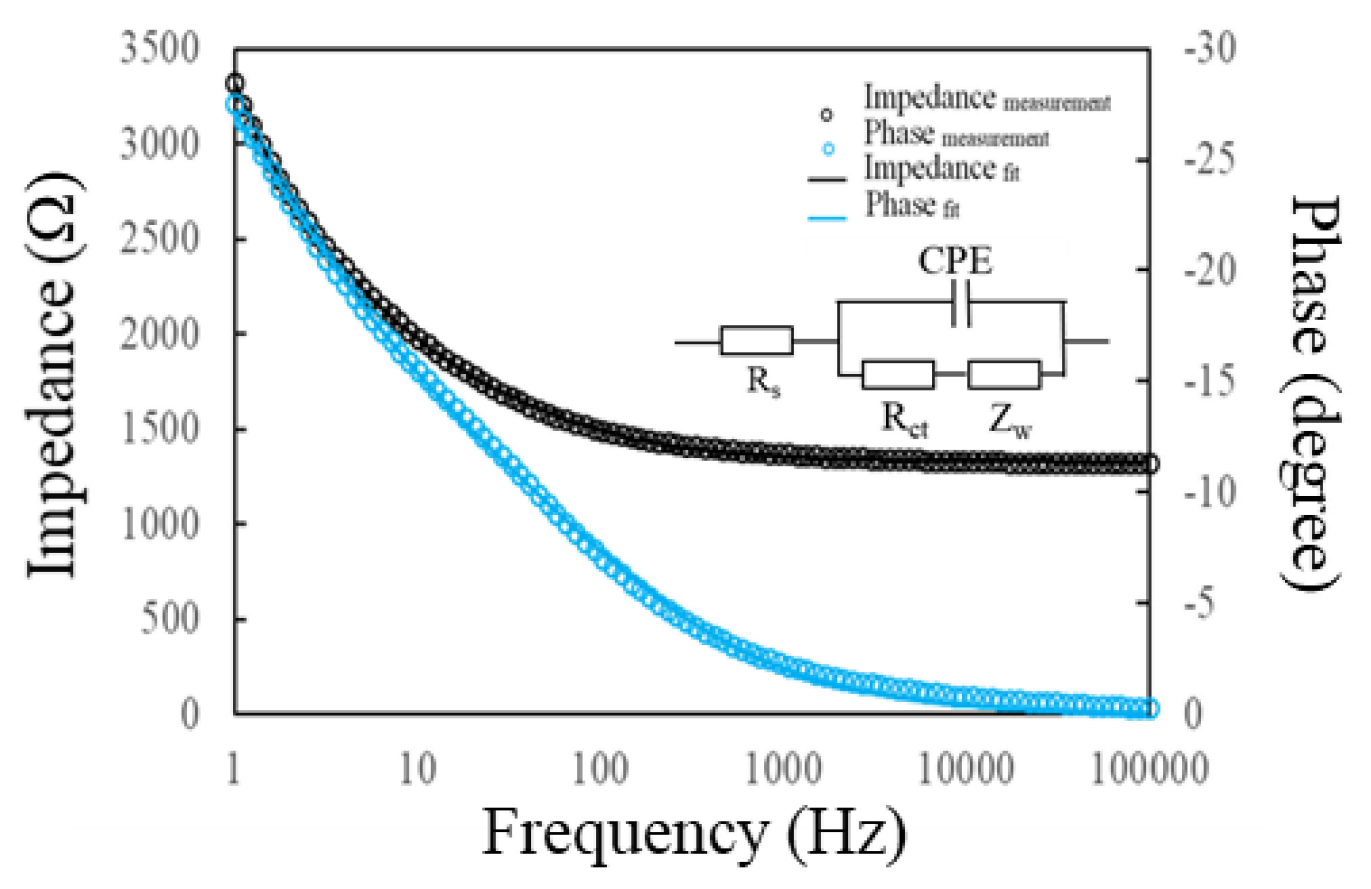
Modified Randles equivalent circuit diagram (inset) used to fit the impedance spectra for the PA(100):BSA(100)/MPA/AuNS/SPCEs. Black and blue circle points indicate the experimentally measured impedance and phase angle values, respectively. Black and blue lines show the corresponding fitting results.

The Randles equivalent circuit, consisting of four elements—the solution resistance (*R_s_*), the Warburg impedance (*Z_w_*), the pure capacitance of electrical double layer (*C_dl_*) and the electron transfer resistance (*R_et_*)—is frequently used to explain the diffusive and kinetic behavior of solution/electrode interfaces. As the modified MPA molecules and immobilized PA/BSA proteins have difficulty forming a dense and pinhole-free modification layer on the rough AuNS, the constant phase element (*CPE*) was used to replace the *C_dl_* to elucidate the inhomogeneity of the electrode surface [9,26]. The modified Randles equivalent model is shown in the inset of Figure 3. The impedance of the *CPE* can be presented by *Z_CPE_(ω) = Z_0_(jω)^−α^*, where *Z_0_* is a constant, *j* is an imaginary number, *ω* is the angular frequency, and 0 < *α* < 1. When *α* is closer to 1, the *CPE* becomes more capacitive. The modified Randles equivalent circuit, using *CPE* instead of *C_dl_,* is more suitable to explain the electrochemical properties of the modified electrodes [27]. Figure 3 shows the Bode plots for the PA(100):BSA(100)/MPA/AuNS/SPCE, obtained from computer fitting using the modified Randles equivalent circuit and the experimental measurement. The fitting result shows a good consistency with the experimental measurement. The modified Randles circuit model was used to obtain the element values for the AuNS/SPCEs, followed by MPA modification, PA/BSA immobilization and anti-SAL affinity, which have a mean error of less than 0.2% and a maximum error of 2.1% for all fitting data.

In Faradic impedance measurement, the *R_et_* value is more sensitive to the change in the interfacial impedance than the *CPE* value [9,26,28]. Therefore, the *R_et_* value is used to quantitatively evaluate the effect of different PA:BSA modifications on the amount of adsorbed anti-SAL, as listed in Table 1. The *R_et_* values, measured at the AuNS/SPCEs, were only 70−80 Ω, implying that the AuNS deposition supplies a high conductance and a large surface to significantly promote the redox current of Fe(CN)_6_^3−/4−^. After the MPA modification, the *R_et_* values significantly increased to 0.71−0.74 kΩ, which was mainly attributed to the effect of electrostatic repulsion between the carboxylic acid of the MPA and the negatively charged Fe(CN)_6_^3−/4−^ [26,28]. Before the immobilization of the PA:BSA mixture and the anti-SAL adsorption, the *R_et_* values significantly increased due to the steric hindrance of protein macromolecules and the negative charges of the proteins (isoelectric point (pI, 4.7) of BSA, pI (around 5.0) of PA). Calculating the *R_et_* increment (Δ*R_et-PA:BSA_*) of the PA:BSA immobilization can realize the immobilized magnitude of PA and BSA proteins on the surface of EDC/NHS-activated MPA/AuNS/SPCEs, as shown in Table 1. The Δ*R_et-PA:BSA_* values increased with the total concentration of PA and BSA, from 100 to 200 μg/mL. Furthermore, the Δ*R_et-PA:BSA_* of PA(100):BSA(200) immobilization was slightly smaller than that of PA(100):BSA (100) immobilization, decreasing by 3.4%. The results demonstrate that an excessive proteins concentration (300 μg/mL) cannot increase the surface coverage of immobilized proteins, and the total concentrations of 200 μg/mL proteins can produce saturated coverage on the electrode surface.

After the immobilization of PA(100):BSA(0) and PA(100):BSA(50), the coverage ratio of proteins versus the saturated coverage of PA(100):BSA(100) was respectively found to be 83% and 94% by calculating the relative Δ*R_et-PA:BSA_* values. Furthermore, the concentration of 200 μg/mL proteins is assumed to produce 100% coverage, and the PA surface density on the PA(100):BSA(0)-, PA(100):BSA(50)-, PA(100):BSA(100)- and PA(100):BSA(200)-immobilized electrodes are respectively calculated as 83%, 63% (= 94%×(100/150)), 50%(= 100%×(100/200)) and 33%(= 100%×(100/300)) of the saturated protein layer. The *R_et_* increment values (Δ*R_et-anti-SAL_*) of antibody adsorption are listed in Table 1 to quantify the magnitude of adsorbed anti-SAL on the density-varied PA-modified electrodes. The result shows that Δ*R_et-anti-SAL_* increased with the decreasing PA density, in the 83%−50% range. Similarly, Boujday et al. found that a PA layer with about 60% coverage adsorbed more IgG than a PA layer with 100% coverage, as observed by infrared spectroscopy and quartz crystal microbalance [25]. The phenomenon is attributed to the PA molecules having a lower coverage with a greater flexibility and accessibility for antibody adsorption. However, we think that, in addition to contributing to the flexibility of a low coverage PA layer, the ordered arrangement of PA molecules also plays an important role in the antibody adsorption. In principle, the *R_et_* measured on an immunosensor is affected by the thickness, structure and polarity of the biorecognition layer. It is difficult to realize the actual orientation of the adsorbed anti-SAL by using EIS measurement. The amount and orientation of adsorbed anti-SAL determines the thickness and the structure of the anti-SAL/PA:BSA biorecognition layer. The relationship between the *R_et_* value and the amount/structure parameters of anti-SAL/PA:BSA may be represented by a linear regression or an exponential regression. According to the results in Table 1, the Δ*R_et-anti-SAL_* didn’t show a linear relationship with the PA density, because the Δ*R_et-anti-SAL_* obtained from the 33%PA density of PA(100):BSA(200)-immobilized electrode is much lower than that obtained from the 50%PA density of PA(100):BSA(100)-immobilized electrode. It is worth studying the effect of PA/BSA ratio on the amount of adsorbed antibodies by using different analytical techniques.

Furthermore, the PA(100):BSA(100) immobilization produces a saturated and compact protein layer, which may reduce the PA flexibility, but the 50% PA density of PA(100):BSA(100)-modified electrodes had a significantly higher Δ*R_et-anti-SAL_* (*p*<0.05) than other PA density-modified electrodes, evaluated by Student’s *t*-test. The result implies that the 50% density PA molecules of the ordered and compact arrangement help absorb more antibodies than the more flexible PA layer with 83% density, because the 83% coverage PA layer had a shorter interval between different PA molecules and a more flexible structure, which caused the adsorbed antibodies to tilt and produce steric hindrance, thus reducing the amount of antibodies adsorbed. Insets of Figure 2a–c respectively show antibody adsorption schemes on the protein layers with 83%, 63% and 50% density PA. Furthermore, the electrodes with 33% density PA had the lowest Δ*R_et-anti-SAL_*, indicating that a reduced PA density correlates with lower amounts of antibodies adsorbed, as the PA is immobilized in a compact protein layer. The corresponding scheme is depicted in the inset of Figure 2d. These results suggest that both the density and flexibility of immobilized PA molecules determine the numbers and orientation of the adsorbed antibodies.

### 3.3. Calibration Curves 

**Figure 4:**
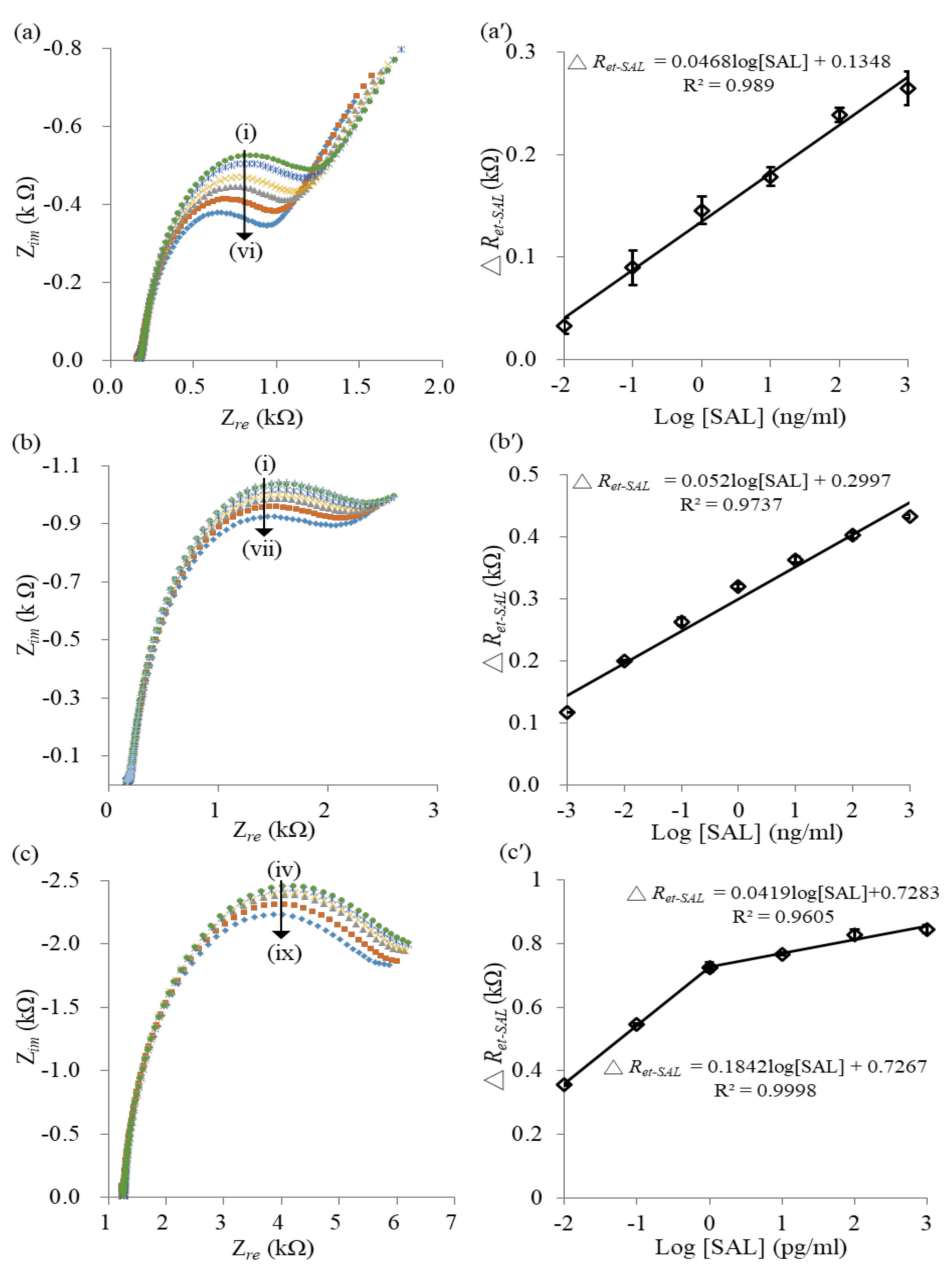
Impedance spectra of the anti-SAL/MPA/AuNS/SPCE- (**a**), the anti-SAL/PA(100):BSA(0)/MPA/AuNS/SPCE- (**b**) and the anti-SAL/PA(100):BSA(100)/MPA/AuNS/SPCE-based (**c**) immunosensors interacting with concentration-different SAL samples. Curves (i)−(ix) respectively represent 1 μg/mL, 100 ng/mL, 10 ng/mL, 1 ng/mL, 100 pg/mL, 10 pg/mL, 1 pg/mL, 100 fg/mL and 10 fg/mL SAL. (**a**′−**c**′) are the calibration curves corresponding to (**a**−**c**) (*n* = 3).

The amount of effective paratopes of adsorbed antibody determines the magnitude of antigen binding. In the study, three kinds of immunosensors based on the anti-SAL/MPA/AuNS/SPCEs, the anti-SAL/PA(100):BSA(0)/MPA/AuNS/SPCE and the anti-SAL/PA(100):BSA(100)/MPA/AuNS/SPCEs were compared in terms of their sensing properties. The 0.1 mg/mL anti-SAL was randomly immobilized on the EDC/NHS-activated MPA/AuNS/SPCEs. The Δ*R_et-anti-SAL_* obtained by the computer fitting of the modified Randles equivalent circuit was 0.20 ± 0.01 kΩ, which is smaller than the Δ*R_et-anti-SAL_* (0.75 kΩ) of anti-SAL/PA(100):BSA(0)/MPA/AuNS/SPCE. The result indicates that the PA layer can increase the amount of immobilized antibodies. Figure 4a shows the impedance spectra, measured at the anti-SAL/MPA/AuNS/SPCEs for concentration-varied SAL samples. All Nyquist plots obtained from the anti-SAL/MPA/AuNS/SPCEs presented an obvious linear part and a small semicircular part, indicating the low interfacial impedance required to produce the significant diffusion-controlled behavior due to the smaller amount of randomly oriented anti-SAL [26]. Moreover, the semicircle’s radius increased with the SAL concentrations, implying increased interfacial impedance. After fitting the experimental spectra by using the modified Randles circuit, the relationship between the Δ*R_et-SAL_* and the SAL concentration is shown in Figure 4a. The calibration curve obtained from three individual electrodes had a linear regression equation of Δ*R_et-SAL_* (kΩ) = 0.0468 log[SAL] + 0.1348 with a linear range of 10 pg/mL to 1 μg/mL. The calculated LOD was 10 pg/mL (S/N > 3).

Figure 4b displays the impedance spectra, measured at the anti-SAL/PA(100):BSA(0)/MPA/AuNS/SPCEs for the immunoreaction of concentration-varied SAL samples. The Fc region of the anti-SAL can be specifically adsorbed on the PA-modified electrodes to produce an oriented arrangement. Moreover, the Nyquist plots of SAL immunoreaction, measured at the anti-SAL/PA(100):BSA(0)/MPA/AuNS/SPCEs, show a smaller linear region and a larger semicircular region than those measured at the anti-SAL/MPA/AuNS/SPCEs, implying that the immobilized PA and the oriented anti-SAL produce a more compact protein layer on the electrodes to increase the interface impedance. The experimental spectra were fitted by a modified Randles circuit to obtain the Δ*R_et-SAL_*. The corresponding calibration curve is shown in Figure 4b, which had a linear regression equation of Δ*R_et-SAL_* (kΩ) = 0.0521 log[SAL] + 0.2997 with a linear range of 1 pg/mL to 1 μg/mL. The calculated LOD was 0.1 pg/mL (S/N>3). The larger sensitivity of the calibration curve and lower LOD indicates the anti-SAL/PA(100):BSA(0)/MPA/AuNS/SPCEs have more effective paratopes than the randomly immobilized anti-SAL/MPA/AuNS/SPCE-based immunosensors.

Figure 4c shows the Nyquist plots of the SAL immunoreaction, measured at the anti-SAL/PA(100):BSA(100)/MPA/AuNS/SPCEs. Compared to the previous two immunosensors, the anti-SAL/PA(100):BSA(100)/MPA/AuNS/SPCEs had more adsorbed anti-SAL. The Nyquist plots show the negligible linear region and the significant semicircular region, implying a high degree of interfacial impedance due to the compact protein layer of PA(100):BSA(100) and greater anti-SAL adsorption. The EIS results can be explained using the 1R//C model, consisting of *R_s_* in series with one parallel circuit comprising a *R_et_* and a *CPE* [27]. The calibration curve of the immunosensors is shown in Figure 4c, which is presented as two linear regression equations: Δ*R_et-SAL_* (kΩ) = 0.0419 log[SAL] + 0.7283, with a linear range of 1 ng/mL to 1 pg/mL and Δ*R_et-SAL_* (kΩ) = 0.1842 log[SAL] + 0.7267 with a linear range of 1 pg/mL to 10 fg/mL. The sensitivity of the anti-SAL/PA(100):BSA(100)-modified immunosensors measured for the lower concentrations was much higher than for the higher concentrations, implying the effective paratopes of oriented anti-SAL with high accessibility and immunoreacting affinity with SAL of lower concentration. The phenomenon is similar to the results of Hafaiedh’s study, which reported protein G-modified immunosensors with a significantly increased sensitivity in the C-reactive protein (CRP) detection of low concentrations compared to the random physisorption of anti-CRP-modified immunosensors [24]. The calculated LOD was 0.2 fg/mL (S/N>3) which is much lower than that obtained at the anti-SAL/MPA/AuNS/SPCEs and the anti-SAL/PA(100):BSA(0)/MPA/AuNS/SPCEs. The result proves that the anti-SAL adsorbed on a protein layer of 50% density PA has the more effective paratopes to obtain a more sensitive immunoreaction and a lower LOD. Moreover, the relative standard deviation (<2.5%) of Δ*R_et-SAL_* for each SAL measurement obtained at the anti-SAL/PA(100):BSA(100)/MPA/AuNS/SPCE-based immunosensors (*n* = 3 electrodes) is significantly smaller than that (<23.4%) of the Δ*R_et-SAL_* measured at the anti-SAL/MPA/AuNS/SPCE-based immunosensors (*n* = 3). The result implies that the protein layer with a 50% PA density of constructs a more compact and stable sensing interface on the sensor surface to promote reproducibility. Moreover, the label-free anti-SAL/PA(100):BSA(100)/MPA/AuNS/SPCEs-based immunosensors had a lower LOD than other reported SAL immunosensors with sandwich and competitive immunoassay methods, as compared in Table 2 [26,29,30,31]. This implies that the anti-SAL/PA:BSA/AuNS/SPCEs have promise to act as highly sensitive immunosensors for the detection of small molecule chemicals.

The monoclonal anti-SAL used in the literature [26] and this work is a monovalent antibody, which is suitable for label-free detection. If the antibodies are required for sandwich immunoassay [29], the paratope number of antibodies should be larger than 1.

### 3.4. Other Sensing Properties

In real sample measurement, matrix interference is an unavoidable challenge for label-free impedimetric immunosensors. Although the matrix influence can be reduced or eliminated by diluting real samples [34,35], the analyte concentration is simultaneously diluted. Therefore, a high sensitivity and low LOD immunosensor is needed for the dilution sensing strategy. An SAL of 0.01, 0.1 and 1 ng/mL was spiked in a commercial porcine serum to estimate the practical application of the immunosensors. After diluting the SAL-containing serum by a factor of 1000 with 10 mM PBS to the 0.01, 0.1 and 1 pg/mL SAL concentrations, the Δ*R_et-SAL_* values were measured and calculated using the regression equation, as shown in Table 3. The recovery of detected 0.01−1 pg/mL SAL was in the range of 95.1–99.0%. The result shows a high feasibility for using the anti-SAL/PA(100):BSA(100)/MPA/AuNS/SPCEs to develop a commercial immunosensor for the practical measurement of SAL concentrations in real samples. Moreover, the selectivity of the developed immunosensor was investigated from the individual responses to 1 ng/mL ractopamine (RAC), clenbuterol (CLB) and SAL. The ratios of Δ*R*_et*-RAC*_ and Δ*R*_et-*CLB*_ to the Δ*R*_et-*SAL*_ were, respectively, 6.25% and 3.27% (data not shown). The result implies that the immunosensors have good specificity and selectivity.

## 4. Conclusions

The mixture of PA and BSA was used to modify the EDC/NHS-activated MPA/AuNS/SPCEs for the oriented immobilization of anti-SAL. Particularly, the PA/BSA protein layer constructed by the concentrations of 100 μg/mL PA and 100 μg/mL BSA can supply greater adsorption of monoclonal anti-SAL, which can improve the sensing properties of EIS-based immunsensors. The anti-SAL/PA(100):BSA(100)/MPA/AuNS/SPCE-based immunosensors present an ultrasensitive detection for SAL, with an extremely low LOD (0.2 fg/mL) and good recovery for the practical measurement of SAL in 1000-times-diluted porcine serum samples. The PA/BSA protein layer with 50% PA density has great promise for greater adsorption of different IgG antibodies to construct highly sensitive immunosensors.

## Figures and Tables

**Table 1:** Values of the *R_et_* element respectively fitted from the experimental spectra of Figuew 2a–d and the calculated Δ*R_et-PA:BSA_*(= *R_et-PA:BSA_* − *R_et-MPA_*) and Δ*R_et-anti-SAL_*(= *R_et-anti-SAL_* − *R_et-PA:BSA_*). (*n* = 3)

Modification	*R_et_* (kΩ) of Different PA:BSA-Modified Electrodes			
	100:0	100:50	100:100	100:200
AuNS	0.07 ± 0.00	0.08 ± 0.00	0.08 ± 0.00	0.08 ± 0.00
MPA	0.71 ± 0.01	0.72 ± 0.01	0.74 ± 0.01	0.72 ± 0.01
PA:BSA (μg/mL)	2.68 ± 0.06	2.94 ± 0.02	3.10 ± 0.06	3.00 ± 0.06
Anti-SAL	3.43 ± 0.01	3.77 ± 0.01	4.08 ± 0.01	3.14 ± 0.02
Δ*R_et-PA:BSA_*	1.97 ± 0.06	2.22 ± 0.02	2.36 ± 0.01	2.28 ± 0.06
Δ*R_et-anti-SAL_*	0.75 ± 0.06	0.83 ± 0.01	0.98 ± 0.01	0.14 ± 0.02

**Table 2:** Comparison of the sensing properties of different immunosensors for the detection of SAL.

Electrodes	Methods	Linear Range	LOD	Ref.
Anti-SAL/AuNS/SPCE	Label-free EIS	0.1 pg/mL–1 μg/mL	4 fg/mL	[26]
Anti-SAL/SDBS-GS/GCE^a^	Sandwich immunoassay with Pd@SBA-15^b^/Ab2 by amperometry	0.02–15.0 ng/mL	7 pg/mL	[29]
SAL/rGO/SPCE	Competitive immunoassay with AgPd @anti-SAL by LSV^b^	0.01–100 ng/mL	1.4 pg/mL	[30]
Aptamer/AuE	Label-free DPV^c^ and EIS	0.1–10 pg/mL	0.5 pg/mL	[31]
rGO/AuNPs	Anti-SAL magnetic bead purification by surface-enhanced Raman spectroscopy	1-20 ng/mL	0.5 ng/mL	[32]
--	Europium-labeled goat anti-mouse IgG by time-resolved fluoroimmunoassay	0.014–2.32 ng/mL	0.014 ng/mL	[33]
Anti-SAL/PA:BSA/AuNS/SPCE	Label-free EIS	10 fg/mL–1 ng/mL	0.2 fg/mL	This work

^a^ SDBS-GS/GCE: sodium dodecylbenzene sulfonate-functionalized graphene sheets/glassy carbon electrode; ^b^ LSV: linear sweep voltammetry; ^c^ DPV: differential pulse voltammetry

**Table 3:** The practical measurement of SAL concentration prepared in 1000-times-diluted porcine serum samples. Each concentration was measured by four sensors.

[SAL] (fg/mL)		Recovery (%)
Added	Detected	
10	9.5 ± 0.7	95.1
100	95.7 ± 9.7	95.7
1000	989.7 ± 44.7	99.0
